# Soft Liquid Metal-Based Conducting Composite with Robust Electrical Durability for a Wearable Electrocardiogram Sensor

**DOI:** 10.3390/polym14163409

**Published:** 2022-08-20

**Authors:** Yewon Kim, Jihyang Song, Soojung An, Mikyung Shin, Donghee Son

**Affiliations:** 1 Department of Electrical and Computer Engineering, Sungkyunkwan University, Suwon 16419, Korea; 2 Center for Neuroscience Imaging Research, Institute for Basic Science (IBS), Suwon 16419, Korea; 3 Department of Superintelligence Engineering, Sungkyunkwan University, Suwon 16419, Korea; 4 Department of Intelligent Precision Healthcare Convergence, Sungkyunkwan University, Suwon 16419, Korea; 5 Department of Biomedical Engineering, Sungkyunkwan University, Suwon 16419, Korea

**Keywords:** liquid metal, composite, electrical durability, soft electronics, wearable device

## Abstract

Liquid metals not only have the electrical property of conductivity, but they also have a unique characteristic of existing in a liquid state at room temperature, unlike ordinary stiff solid metals. However, in bioelectronics, the modulus matching well between a device and skin or tissue is considered very advantageous, because high-quality biological signals can be recorded. Therefore, it is possible to implement soft electronics with stable and robust electrical characteristics by using LM as a conductive liquid-state filler. In this study, we changed a type of liquid metal, Eutectic Gallium Indium (EGaIn), into a particle form via tip sonication and mixed it with a solution that dissolved Styrene-Ethylene-Butylene-Styrene (SEBS) in toluene to fabricate a composite. The EGaIn-SEBS composite has high conductivity, excellent electrical durability under mechanically harsh conditions, and a degree of modulus similar to that of bare SEBS, which is lower than that of solid-filler-based SEBS composite. Finally, we demonstrated electrocardiogram signal monitoring using an EGaIn-Alginate two-layer electrode (EATE) that was fabricated by simply coating the surface of the composite with alginate hydrogel, which demonstrates excellent performance in bioelectronics.

## 1. Introduction

Recently, the importance of soft and stretchable bioelectronics has been increasing. Such bioelectronics have the advantages of being well matched with the modulus of skin and tissue and not causing any mechanical damage to human body [1,2,3,4,5,6]. To realize these characteristics, two methods are typically used in bioelectronics. The first method involves using a buckled, wavy, and serpentine design to provide structural stretchability to devices [7,8,9,10]. The second method involves forming a composite by mixing conductive fillers in a stable and soft polymer [11,12,13,14,15]. In this case particularly, metal-based materials are widely used as conductive fillers, and conductivity can be easily ensured using a solid-state metal. However, due to the brittleness and stiffness of solid-state metal fillers, the composite shows poor mechanical performance, for example, considerably lower stretchability than the original stretchability of polymer or a significant increase in the modulus [16]. Therefore, if high conductivity can be ensured and mechanical properties, such as softness and stretchability, can be maintained better through a simple process of mixing with conductive materials and polymers, the resulting conductor can be an excellent material for soft bioelectronics.

A liquid metal (LM) is a remarkable material capable of overcoming the aforementioned trade-off between electrical and mechanical properties. LMs have fluid-like properties, such as negligible vapor pressure (close to zero), low melting point, and high surface tension [17,18,19]. Moreover, LMs have high thermal and electrical conductivity [20,21]. Thus, LMs show mechanical properties similar to liquids under room temperature, while also having metallic characteristics. Using these unique advantages of LMs, conductors, electrodes, interconnects, antennas, soft sensors, and actuators have been studied using various methods such as injecting [22,23], direct writing [24,25], laser patterning [26,27], and micropatterning [28,29]. The majority of the research shows the possibility of self-healing using the property that, unlike solids, liquids merge again when they come into contact with each other. In addition, an LM is changed into a particle form and mixed with a soft polymer to fabricate the composite [30,31,32,33,34] which has the advantage of maintaining the softness of the polymer considerably better because the inside of the particle is still fluid. Furthermore, many studies have proved that a Ga-based LM has biocompatibility, that is, it is harmless to human body [35,36]. Therefore, a Ga-based LM is appropriate to be used as a material for electrodes and interconnections of soft bioelectronics.

**Figure 1:**
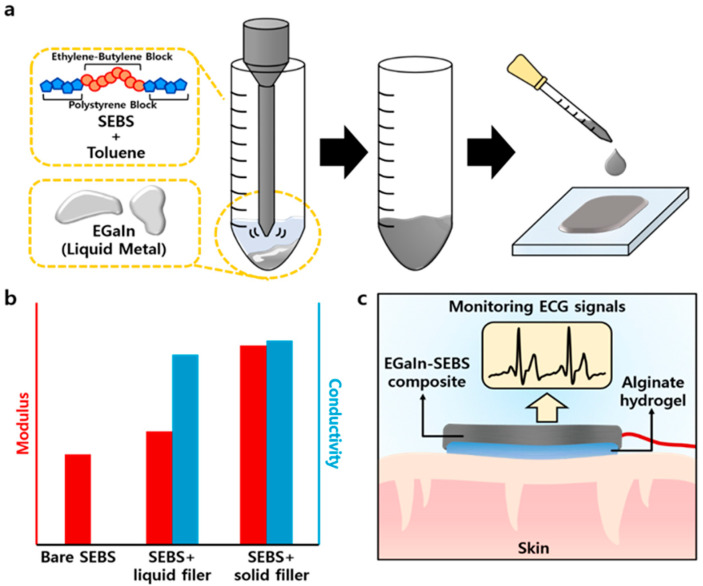
(**a**) Schematic of the tip sonication process fabricating the EGaIn-SEBS composite. (**b**) Bar graph comparing electrical and mechanical properties among the bare SEBS, liquid filler composite, and solid filler composite. (**c**) Schematic illustration of ECG sensor.

In this study, we used Eutectic Gallium Indium (EGaIn), one of the most commonly used Ga-based LMs, and stretchable thermoplastic elastomer Styrene-Ethylene-Butylene-Styrene (SEBS) to demonstrate wearable soft bioelectronics for electrocardiogram (ECG) monitoring. We split the conditions according to the amount of EGaIn and changed it to a particle form via tip sonication. Subsequently, we mixed it with toluene in which SEBS was dissolved, drop-casted it on the glass substrate, and dried it under room temperature. The optimized EGaIn-SEBS composite showed excellent electrical properties with high initial conductivity and without an increase in resistance (ΔR/R_0_~−1.67%) under harsh conditions. Additionally, its mechanical properties were largely similar to those of the bare SEBS film, in contrast to when using solid fillers (Ag Flakes) as composites. The EGaIn-SEBS composite showed a much lower modulus than the solid-state filler composite and better mechanical recoverability. Finally, we used an EGaIn-Alginate two-layer electrode (EATE), which was fabricated via alginate hydrogel coating on the surface of the composite, to demonstrate a wearable ECG sensor to accurately measure cardiac signals. The alginate layer improved the both mechanical and electrical properties of the device recording the physiological signals (Figure 1a–c).

## 2. Materials and Methods

### 2.1. Fabrication of Composites

To fabricate the EGaIn-SEBS composite, firstly, 180 mg of SEBS (Tuftec™ H1062, Asahi Kasei Co., Ltd., Tokyo, Japan) was dissolved in 1.5 mL of toluene (Sigma-Aldrich, Burlington, MA, USA). Subsequently, 400 mg, 700 mg, and 1 g of EGaIn (12478, Alfa Aesar, Ward Hill, MA, USA) were added to the solution, and tip sonication (VCX-750, Sonics & Materials Inc., Newtown, Connecticut, USA) was performed for 10 min at 300-W power. The EGaIn-SEBS solution was drop-casted in 2 cm × 2 cm glasses and evaporated at room temperature for 6 h to complete a homogeneous composite. Ag flakes (AgFs, DSF-500MWZ-S, Daejoo Electronic Materials Co., Ltd., Gyeonggi-do, South Korea) were used as solid-state fillers for comparison with EGaIn, which is a liquid-state filler. A total of 700 mg of AgFs was added to the toluene in which SEBS was dissolved, and the AgF-SEBS solution was dispersed well using a vortex mixer (MaXshake™ VM30, Daihan Scientific Co., Ltd., Gangwon-do, South Korea). Thereafter, the AgF-SEBS composite was fabricated in the same way as the aforementioned process. Finally, the microscopic structure and EDS (Energy Dispersive X-Ray Spectrometer) mapping of the composites were analyzed using FE-SEM (JSM-IT800, JEOL Co., Ltd., Tokyo, Japan).

### 2.2. Mechanical and Electrical Characteristics of Composites

To evaluate the electrical properties of the composites, a digital multimeter (Keithley 2450 SourceMeter, Tektronix, Inc., Beaverton, Oregon, USA) and motor-based x-axis stretcher (SMC-100, Jaeil Optical Corp., Daegu, Korea) were used. To measure the changes in strain-dependent electrical resistance, the samples (with initial widths and lengths of 3 mm and 20 mm, respectively) were loaded on the stretcher using double-sided tape (3M, Maplewood, MN, USA); the initial length before conducting the stretching test was 3 mm. The samples were stretched at a rate of 3 mm min^−1^. For the cyclic test, the samples were loaded under the same condition as that of the aforementioned stretching test and stretched from 0% to 50%. This was repeated for 200 cycles at a rate of 20 mm min^−1^.

Next, to evaluate the electrochemical impedance of composites, a potentiostat (ZIVE SP1, WonATech Co., Seoul, Korea) was used. The area of the samples was 0.5 cm × 0.5 cm. The sample, a Ag/AgCl reference electrode, and a platinum counter electrode were immersed in PBS (1X PBS Buffer, pH 7.2, Biosesang, Gyeonggi-do, Korea) to obtain data. The frequency ranges of impedance were 0.1 Hz to 100 kHz, and an amplitude of 10 mV was used.

Finally, to measure the mechanical properties, a universal testing machine (UTM, Instron 34SC-1, Norwood, MA, USA) was used. The widths and lengths of the samples were 5 mm and 20 mm, respectively. When loading the samples on the chuck, the initial length was 5 mm. The speed rate was 20 mm min^−1^, and all measurement data were obtained using Instron Bluehill software.

### 2.3. Electrocardiogram Monitoring Using EGaIn-SEBS Composite with Alginate Hydrogel Layer

Alginate hydrogel was coated on the surface of the EGaIn-SEBS composite to enable it to operate well as a surface electrode of the ECG sensor. To make the hydrogel, 500 mg of sodium alginate (Sigma-Aldrich, MA, USA) was dissolved in 4.5 mL of deionized water (10 wt%). Then, the sodium alginate solution was dropped onto the surface of the composite evenly. Subsequently, the ECG sensor electrode comprising two layers (hydrogel and composite) was fixed to the skin using a Tegaderm film (3M, Maplewood, MN, USA). In total, three 2 cm × 2 cm composites were used as the reference electrode attached to the right hand, ground electrode on the left hand, and active electrode on the left leg. The ECG signals were monitored for 10 min each before and after run. At this time, a bio-signal amplifier (Bio Amp, ADInstruments, Dunedin, New Zealand) and data acquisition device (PowerLab 8/35, AD Instruments, Bella Vista, Australia) were used to collect the signals. LabChart 8 Pro (ADInstruments, Dunedin, New Zealand) software was used to obtain all data. The authors obtained Institutional Review Board (IRB) approval (No. SKKU 2022-07-034) from Sungkyunkwan University for the measurement of the ECG signals.

## 3. Results

### 3.1. Optimization Process of the Composite According to the Weight Ratio of EGaIn

**Figure 2:**
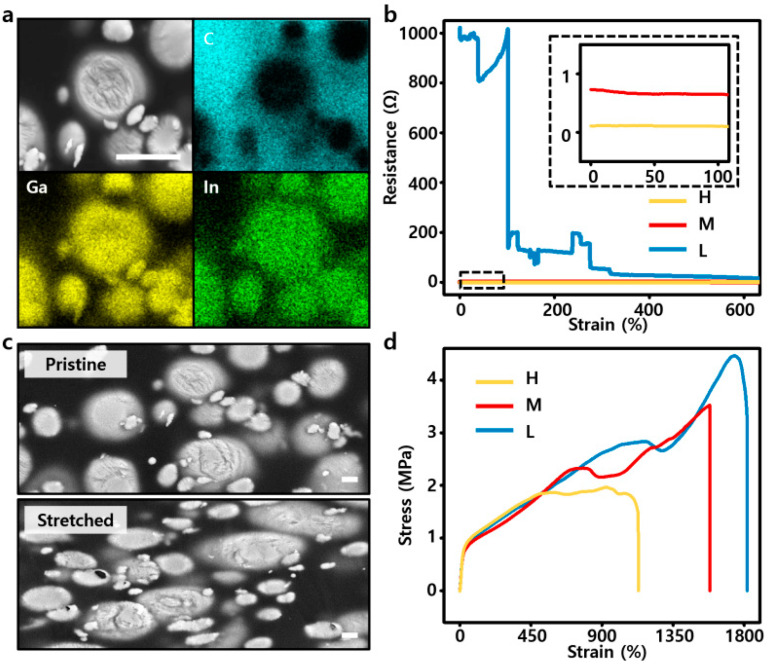
(**a**) SEM and corresponding EDS images of the EGaIn-SEBS composite (scale bar: 5 μm). (**b**) Resistance–strain characteristics of EGaIn-SEBS composites having three conditions according to weight ratios (H, M, and L (meaning high, middle, and low weight ratio of EGaIn in the composite)). (**c**) SEM images of the EGaIn-SEBS composite before being stretched (**top**) and after being stretched (**bottom**) (scale bar: 1 μm). (**d**) Stress–strain curves of the EGaIn-SEBS composites having three conditions according to weight ratios (H, M, and L).

A homogeneous EGaIn-SEBS composite was produced using the tip sonication method by turning the bulk EGaIn in the SEBS solution into a particle form. SEM and EDS mapping images showed that EGaIn particles were well dispersed inside the SEBS matrix (Figure 2a). The amount of SEBS was fixed, and the electrical and mechanical properties of High (H) with 1 g, Middle (M) with 700 mg, and Low (L) with 400 mg of EGaIn were compared according to the weight ratio of EGaIn, which is a conductive liquid-state filler. When the EGaIn-SEBS composites were stretched up to 600%, as shown in Figure 2b, the initial resistance values were 0.11 Ω, 0.73 Ω, and 1020.02 Ω for H, M, and L, respectively. However, these composites had a unique characteristic; the more they were stretched, the lower the electrical resistance was, or it was at least maintained at the same level. Particularly, in the case of L, which had the highest initial resistance value, the resistance was greatly decreased. M and H showed a slight decrease, or nearly maintained the same degree, even though the initial resistance before being stretched was very low. This was because of the liquid-state property of EGaIn. Figure 2c shows the inside of the EGaIn-SEBS composite when it was pristine and stretched. EGaIn particles generally showed a spherical shape when in pristine condition (top), but when stretched, the particles had an oval shape that extended on both sides (bottom). EGaIn particles can maintain their form owing to the oxide layer (Ga_2_O_3_) on the external surface of the particles, even though their inside is still in a liquid state [37,38,39]. Therefore, as the SEBS matrix stretched in one direction, the fluid EGaIn inside the particles flowed in the same direction, stretching the particles themselves. This phenomenon established contact between the separated particles and had a positive effect on the conductivity of the composite. In addition, EGaIn, which is fluid, had mechanical advantages. As shown in Figure 2d, the smaller the amount of EGaIn, the better the sample was stretched; however, H, M, and L had almost the same level of modulus. Therefore, the proportion of the SEBS matrix of the composite also changed relative to the amount of EGaIn, which had a certain effect on its stretchability. However, because EGaIn particles are themselves in a liquid state inside, they have little effect on the composite’s modulus [31,40]. In conclusion, when we compared the three conditions of H, M, and L, L basically had a disadvantage because the initial resistance was very high compared to that of the others, and its electrical resistance was very unstable depending on stretchability. H showed the best conductivity, but its stretchability was lower than that of the other samples. Therefore, we chose M as the optimization condition that had intermediate values in all properties.

### 3.2. Electrical and Mechanical Advantages of EGaIn-SEBS Composite Compared with Solid-Filler Composite

**Figure 3:**
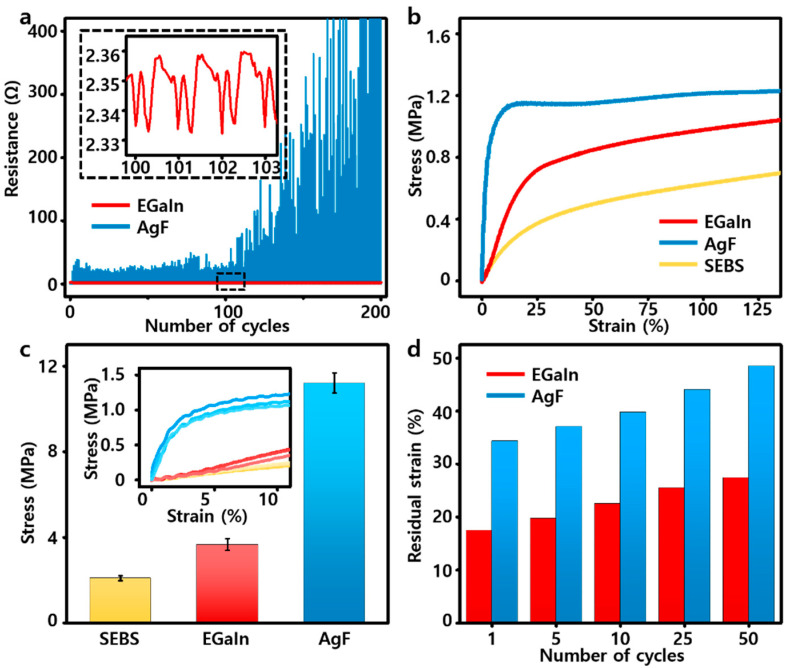
(**a**) Cyclic stretching test of the EGaIn-SEBS and AgF-SEBS composites. (**b**) Stress–strain curves of the SEBS-EGaIn composite, AgF-SEBS composite, and bare SEBS film sample. (**c**) Comparison of the modulus of the samples. Data are expressed as the mean ± s.d. (n = 3) (**d**) Number of cycles dependent on residual strain of the EGaIn-SEBS and AgF-SEBS composites.

To confirm the electrical durability of the EGaIn-SEBS composite, a cyclic test was conducted that repeatedly applied 50% strain to the sample. As shown in Figure 3a, the EGaIn-SEBS composite very stably maintained the electrical resistance value even when the cycles were repeated 200 times. However, in the case of AgF with the same weight ratio in the composite (Supplementary Figure S1), the resistance gradually increased as the cycles were repeated. Additionally, as shown by the stress–strain curve, the EGaIn-SEBS composite had a much lower level of mechanical properties than the AgF-SEBS composite (Figure 3b) even if the weight ratio of the conductive fillers in both composites was same. In particular, the modulus of the AgF-SEBS composite was approximately 11.22 MPa, and those of the bare SEBS and EGaIn-SEBS composites were 2.09 MPa and 3.67 MPa, respectively, which were considerably lower than that of AgF (Figure 3c). The inset image shows the stress–strain curves of all the samples. The aforementioned data clearly showed the difference between solid-state fillers and liquid-state fillers. In the case of solid-state fillers, they were considerably stiffer than the SEBS matrix; therefore, when they were mixed with elastomer and made into a composite, they had a very significant effect on the mechanical properties of the composite. However, in the case of liquid-state fillers, even if the fillers occupied a large weight ratio in the composite, the composite itself barely became rigid [31,40,41]. As shown in Figure 3d, the graph was obtained by repeatedly applying 100% strain to the samples for 50 cycles. The residual strain of the AgF-SEBS composite was higher than the EGaIn-SEBS composite. Additionally, the degree of increase was also greater in the AgF. This means the hysteresis of the EGaIn-SEBS composite was well maintained without fatigue and had much higher mechanical recoverability than the AgF-SEBS composite (Supplementary Figure S2) [11].

### 3.3. Electrocardiogram Monitoring Wearable Device

**Figure 4:**
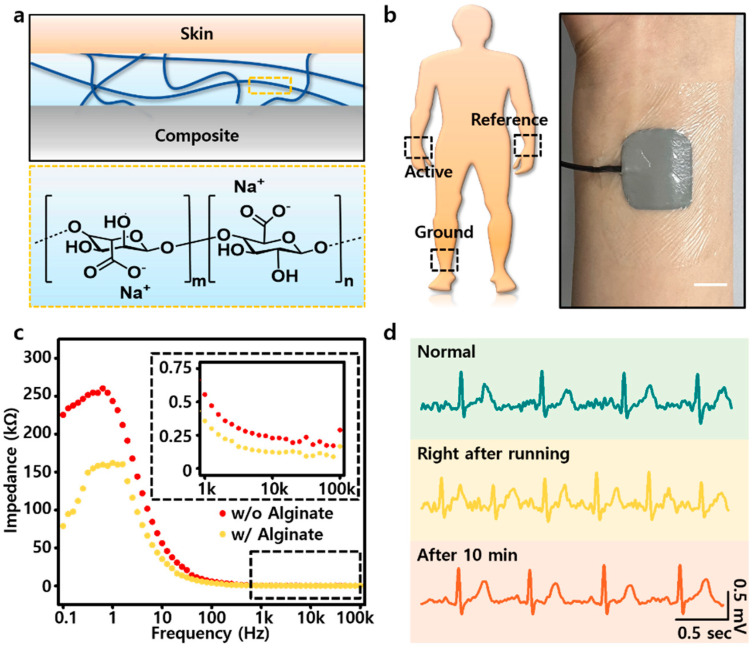
(**a**) Schematic of three layers when EATE is attached to the skin (**top**) and chemical structure of alginate (**bottom**). (**b**) Schematic of electrode positioning for ECG monitoring (**left**) and photograph of EATE (scale bar: 1 cm). (**c**) Impedance of the EATE and EATE without alginate hydrogel (only EGaIn-SEBS composite). (**d**) Real-time ECG signal monitoring before and after running.

Monitoring the biological signals of a human body is a basic and critical factor in diagnosing and judging health conditions and diseases [42]. In particular, if the mechanical characteristics of wearable devices are similar to those of soft human skin, high-quality physiological signals can be obtained without discomfort in wearing them [1,41,42,43]. Therefore, we demonstrated the EGaIn-SEBS composite as a soft ECG sensor electrode capable of recording cardiac signals. EATE (EGaIn-Alginate two-layer electrode) was fabricated with coated alginate solution on the surface of the composite to better match its modulus with that of the skin (Figure 4a, top); it also had ionic conductivity, making it favorable to record cardiac signals. Alginate is found in brown seaweeds and is currently used in many biomedical application fields (Figure 4a, bottom). It has been proved to be suitable for use in soft bioelectronics with high biocompatibility and softness [44,45,46,47]. In EATE, we used 10 wt% of alginate solution on the coating. If the concentration was too high, the alginate would not be dissolved in the solvent uniformly and saturation would occur (Supplementary Figure S3). So, a proper concentration should be maintained. Particularly, since alginate can exhibit its effect even at a lower concentration [47], it is sufficient to maintain a suitable concentration enough to be coated on the surface of the composite. To demonstrate, we attached three EATEs as active, reference, and ground electrodes on the body, as shown in Figure 4b. Additionally, as shown in Figure 4c, EATE had a more stable (lower) impedance than the composite without the alginate layer. This was because of the alginate itself, and metal coordination between metal ions (large amounts of Ga ions and small amounts of In ions) in the oxide layer of EGaIn and carboxylate group in alginate improved the ionic conductivity [48,49,50]. Therefore, this alginate layer not only improved the modulus matching between the skin and the device, but also enabled improved physiological signals to be recorded through this chemical interaction. So, we can monitor the human cardiac signals using EATE. Figure 4d shows the normal cardiac signal before running (top), when the heart rate increases immediately after running (middle), and when the heart rate recovers after a certain period of time (bottom). These demonstration results show that changes in cardiac signals could be accurately monitored using EATE.

## 4. Conclusions

In this study, we demonstrated a soft wearable device for monitoring ECG signals using EATE. Particularly, among the two layers of EATE, the EGaIn-SEBS composite showed excellent conductivity while minimizing the difference in the modulus between the bare SEBS elstomer and the EGaIn-SEBS composite. This could be realized by dispersing the liquid-state EGaIn particles to the SEBS matrix; unlike solid-state fillers mixed composites show rigid mechanical characteristics. Furthermore, EATE with the alginate coating layer added to the surface of the EGaIn-SEBS composite was used for the soft ECG sensor. EATE showed the advantage of minimizing modulus mismatching with the skin as well as having ionic conductivity. Therefore, we believe that EATE is an appropriate soft wearable device for recording physiological signals in a human body.

## Data Availability

Not applicable.

## Supplementary Materials

The following supporting information can be downloaded at: https://www.mdpi.com/article/10.3390/polym14163409/s1, Figure S1: SEM image of AgF-SEBS composite (scale bar: 1 μm), Figure S2: Stress–strain cyclic test of EGaIn-SEBS composite and AgF-SEBS composite, Figure S3: Photographs of 20 wt% and 10 wt% of alginate solution (left) and right after turning over the vials (right) (scale bar: 1 cm).
