# Supplementary material for: Soft Liquid Metal-Based Conducting Composite with Robust Electrical Durability for a Wearable Electrocardiogram Sensor
Source: Polymers (Basel). 2022 Aug 20;14(16):3409. doi: 10.3390/polym14163409 (PMC9416678; doi:10.3390/polym14163409)
Supplement: Supplementary file 1 [file polymers-14-03409-s001.zip › polymers-1871425-supplementary.pdf]

Supplementary Information

Soft liquid metal-based conducting composite with robust electrical durability for a wearable Electrocardiogram sensor

Yewon Kim†, Jihyang Song†, Soojung An, Mikyung Shin*, Donghee Son *

†These authors contributed equally to this work

*Correspondence to be addressed to: *daniel3600@g.skku.edu and mikyungshin@g.skku.edu*

This PDF file includes:

Supplementary Figures. S1–S3

Supplementary Figures

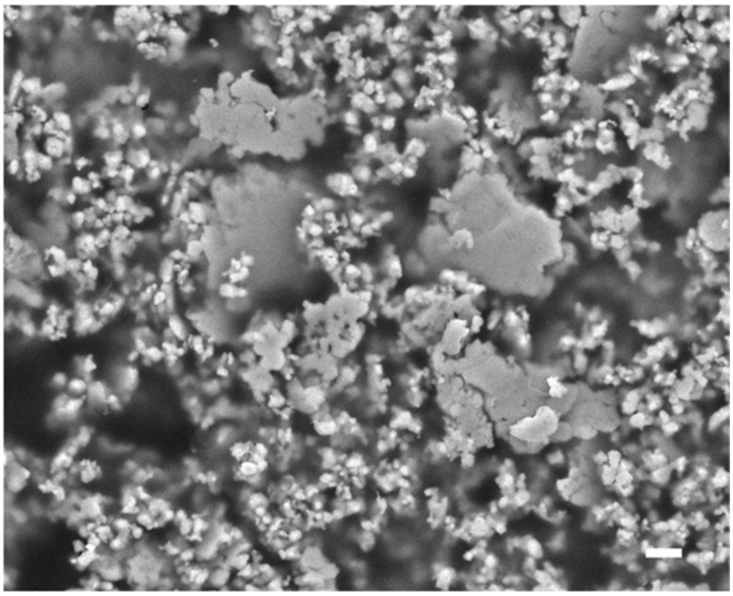

Figure S1. SEM image of AgF-SEBS composite (scale bar : 1 μm).

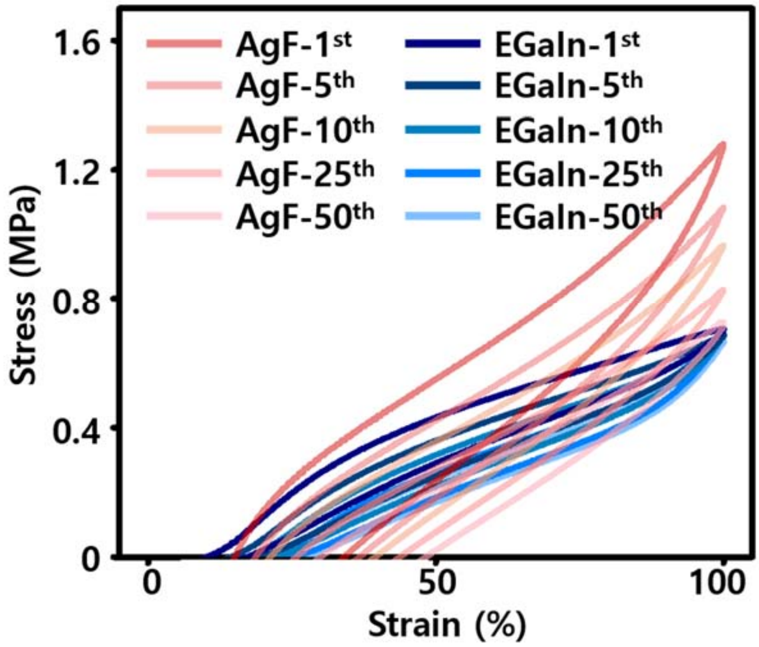

Figure S2. Stress-Strain cyclic test of EGaIn-SEBS composite and AgF-SEBS composite.

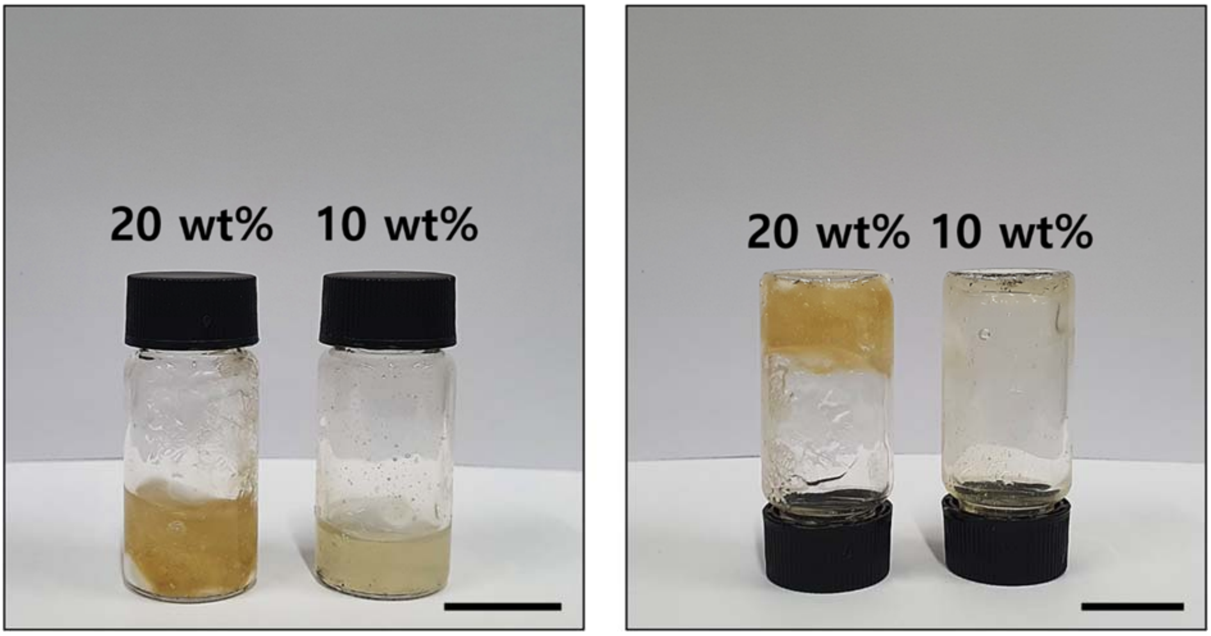

Figure S3. Photographs of 20 wt% and 10 wt% of alginate solution (left) and right after turning over the vials (right) (scale bar : 1 cm).
